# COVID-19 Rekindling Herpes Zoster in an Immunocompetent Patient

**DOI:** 10.7759/cureus.18049

**Published:** 2021-09-17

**Authors:** Piyush Puri, Pankul Parnami, Pal Satyajit Singh Athwal, Sima Kumari, Chandan Kumar, Yogita Suri

**Affiliations:** 1 Internal Medicine, Rama Medical College Hospital & Research Center, Hapur, IND; 2 Paediatrics, Jawaharlal Nehru Medical College, Belagavi, IND; 3 Internal Medicine, Saint Agnes Medical Center, Fresno, USA; 4 Internal Medicine, Patna Medical College, Patna, IND; 5 Gastroenterology, All India Institute of Medical Sciences, Bhubaneswar, IND; 6 Surgery, Saraswathi Institute of Medical Sciences, Hapur, IND

**Keywords:** covid 19, covid, herpes simplex, shingles, covid and skin

## Abstract

During the coronavirus 2019 (COVID-19) pandemic, sundry dermatological conditions related to COVID-19 pneumonia have been published. COVID-19 primarily affects the respiratory system, but secondarily it also affects the heart, kidney, brain, skin, spinal cord, etc. Herpes Zoster (HZ) is considerably important morbidity associated with COVID-19 pneumonia. Recrudescence of HZ occurs because of the latent varicella-zoster virus (VZV) predominantly because of the decline in cell-mediated immunity (CMI). Abating CMI is due to the increasing age, but could also occur if the patient is suffering from an immunosuppressive disease or is using immunosuppressive drugs. In our case, the patient had no lymphopenia unlike the other cases, yet still, he developed HZ. HZ is associated with post-herpetic neuralgia (PHN), HZ ophthalmicus (HZO), and cerebral arteritis increasing morbidity and mortality, especially in elderly people and those who are immunocompromised.

## Introduction

Herpes Zoster (HZ) is a viral skin infection caused by the varicella-zoster virus that remains dormant in dorsal root ganglia following a primary chickenpox infection. People who experience shingles (HZ) during the pandemic can be positive for COVID-19. Dermatological events in patients with COVID-19 include acroischemia, chilblain-like rash, petechiae and purpura, vesicles, hives, and erythematous maculopapules [1]. While COVID-19 is known to have an impact on the immune system and can increase the risk of shingles, limited reports support an association between shingles and COVID-19. 

Atypical presentations of HZ have been published especially in patients who are lymphopenic. HZ could also serve as an indicator of latent COVID-19 infection. Infection with COVID-19 can cause changes in leukocyte levels, resulting in reduced cell counts, mainly CD4+T cells, CD8+T cells, B cells, and natural killer cells [2]. Dermal lesions continue to emerge each day as a marker or complication of COVID-19. Also, SARS-COV2 causes a hyperinflammatory state and the resultant immune dysregulation is also assumed to be the potential cause of reactivation [3]. A lower CD4 count or a disturbance in cell function appears to lead to a gloomier prognosis [4]. People who are immunocompromised or have HIV are at a higher risk of developing the type of necrotic zoster. However, the incidence of this type in healthy individuals indicates a decline in immune cells, B and T lymphocytes, and natural killer cells due to COVID-19 [5].

Unlike the reported data, our patient did not have any lymphopenia or immunosuppression from any cause and still, the patient developed shingles which is quite intriguing.

## Case presentation

**Figure 1:**
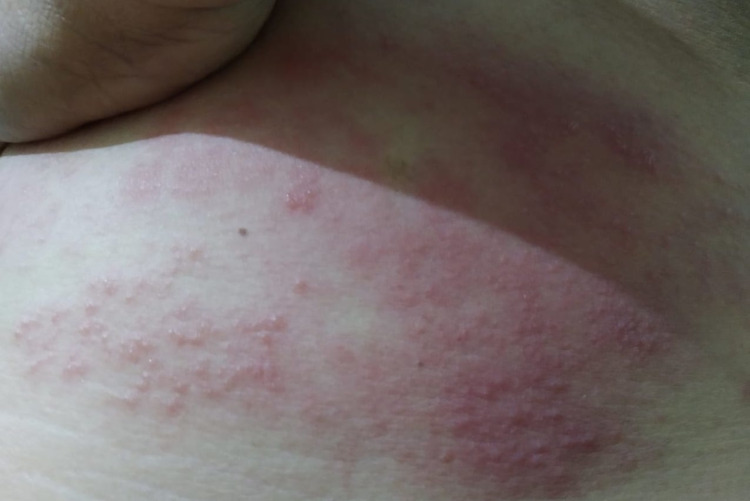
Multiple vesicular lesions affecting in dermatomal distribution.

A 44-year-old male patient consulted the hospital via teleconsultation during the lockdown. Past medical history was not significant except for chickenpox during his childhood. He is not a smoker and a non-alcoholic. He complained of headaches, fever, dry cough, and fatigue for 4 days. The fever was sudden in onset, without any chills and rigors which subsided with the use of acetaminophen. After 2 days of fever, the patient developed itching on the trunk on the left side which revealed lesions with a red erythematous base in a water drop layout. The lesions affected only one side of the body in a dermatomal distribution. Multiple red erythematous vesicular lesions (Figure 1).

**Figure 2:**
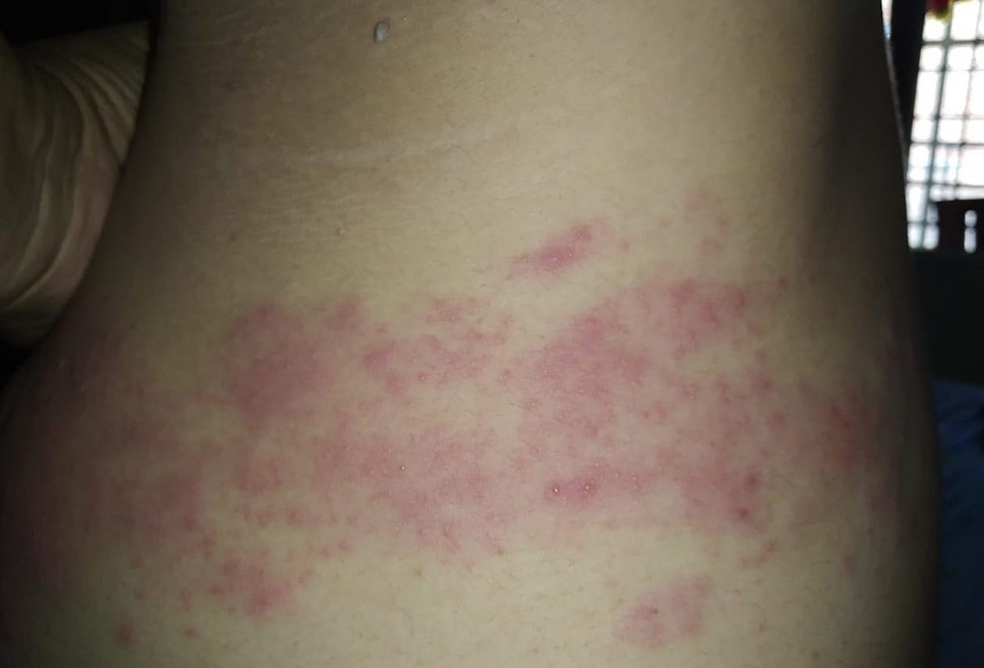
Lesions of HZ spreading from back to the front in a dermatomal fashion. HZ: Herpes Zoster.

Extension of the lesion all the way from the back to the mid-axillary line till the front (Figure 2).

**Figure 3:**
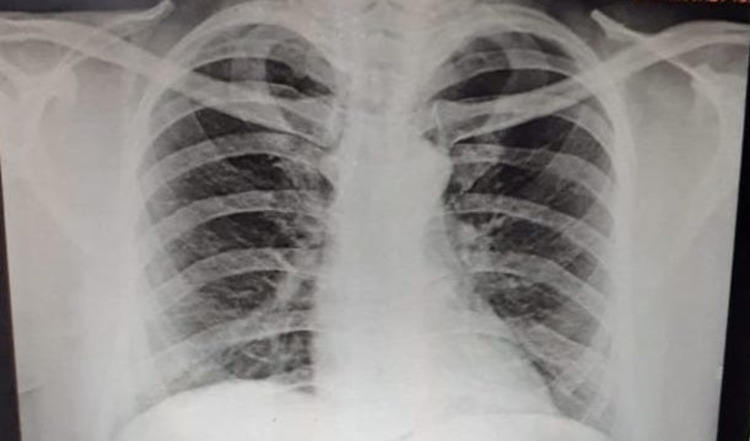
Chest X-ray showing diffuse patchy ground glass opacities in the bilateral lung fields.

RT-PCR for COVID-19 was advised which turned out to be positive and subsequently he was advised of routine blood investigations and a chest X-ray (Figure 3). 

A diagnosis of HZ was made based on the clinical features and the other tests could not be performed because of the country-wide lockdown and the patient avoided any extra exposure. He was treated symptomatically with acetaminophen, pregabalin, and valacyclovir for 10 days. Steroids were avoided for the treatment of COVID-19 considering the risk of a flare-up of HZ.

CBC was done in which the lymphocyte count turned out to be within the normal range, there was no lymphocytopenia, unlike the other published cases (Table 1). 

**Table 1:** Complete blood count had normal lymphocytic count. RBC: red blood cell; WBC: white blood cell; MCHC: mean corpuscular hemoglobin concentration; MCH: mean corpuscular hemoglobin; MCV: mean corpuscular volume; RDW-CV: red blood cell distribution width coefficient of variation; MPV: mean platelet volume; PDW-CV: platelet distribution width coefficient of variation; PCT: platelet crit; PLCR: platelet-large cell rate.

Complete blood count	Finding	Reference range
Haemoglobin	12.6gm/ dl	11-16.5
Total RBC	5.15 mill/cmm	3.8-5.8
Pack cell volume	38.57%	30-50
MCHC	32.9%	30-50
MCH	24.7 pg	26.5-33.5
MCV	75.0 fl	80-99
Total WBC count	7,170/cmm	4,000-11,000
Neutrophils	60%	40-70
Lymphocytes	33%	20-36
Eosinophils	6%	01-06
Monocytes	1%	02-08
Basophils	0%	00-02
Total platelets count	94,000/cmm	1.5-4.5
Absolute lymphocyte count	2.420/uL	0.600-4.100
Absolute neutrophil count	4.320/uL	2.000-7.800
RDW-CV	18.3%	10-15
MPV	12.9%	07-11
PDW-CV	45.2%	37.8-43.6
PCT	0.12%	0.10-0.50
PLCR	59.20%	13-43

The liver function test showed mild derangement mostly due to direct liver injury (Table 2).

**Table 2:** LFT with mild derangements. SGPT: serum glutamic pyruvic transaminase; SGOT: serum glutamic oxaloacetic transaminase; LFT: liver function test.

Liver function test	Finding	Reference range
Total S. Bilirubin	0.6 mg/dL	0.2-1.0
SGPT	46 IU/L	5-35
SGOT	27 IU/L	8-40
S. Alkaline Phosphatase	89 IU/L	37-147
Total protein	6.3 gm/dL	6.0-8.0
Albumin	4.2mg/dL	3.5-5.0
Globulin	2.1 mg/dL	2.3-3.5

The kidney function test had minor derangement (Table 3).

**Table 3:** Kidney function test showing minor increase in serum uric acid.

Kidney function test	Finding	Reference range
Blood urea	22 mg/dL	20-40
Serum uric acid	8.0 mg/dL	3.4-6.7
Serum creatinine	0.9 mg/dL	0.9-1.4

**Figure 4:**
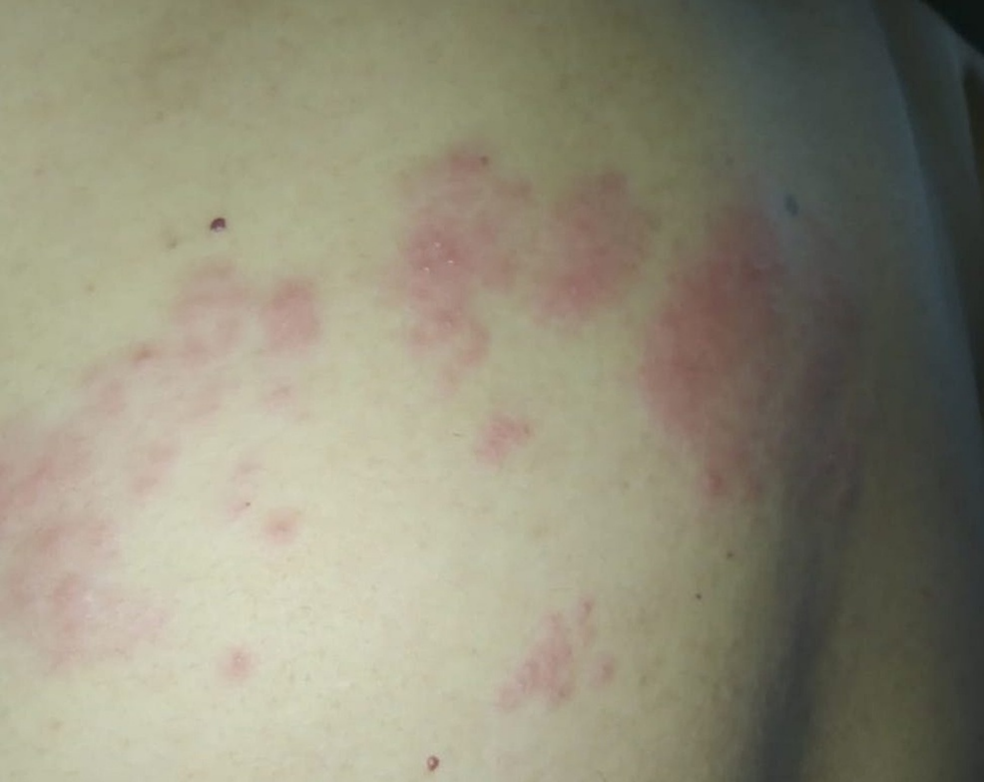
Resolution of Herpes Zoster lesions.

After 10 days of antiviral treatment, there was resolution in the symptoms and the lesions were decreasing (Figure 4). With complete cessation of cough and fever on the 18th day, RT-PCR for COVID-19 was repeated again which was negative and the lesions of HZ had disappeared too.

## Discussion

Severe acute respiratory disease virus-2 (SARS -CoV-2), or COVID -19, has infected more than 200 million people and has led to more than 4.3 million deaths worldwide as of 13th August 2021 [6]. Although many remote cases of herpes zoster and COVID-19 confections have been reported, we present a rare case of herpes zoster infection that manifested during COVID -19 without the presence of lymphopenia. A study conducted in Brazil showed an increase corresponding to an extra 10.7 cases per million inhabitants during the pandemic of HZ which could serve as an indicator of latent COVID-19 infection [7].

Because survivors of COVID-19 can experience enormous psychological stress, patients with COVID-19 may be at risk for shingles as a result of increased psychological stress. T cells have an impact on cell-mediated immunity. When the host's cell-mediated immunity declines, as it does in people with immunodeficiency, HZ is reactivated. COVID19, on the other hand, reduces cell-mediated immunity by lowering lymphocyte counts and decreasing CD3+, CD4+, and CD8+ T cells. 3 As a result, COVID19 may raise the risk of HZ by lowering cell-mediated immunity. Furthermore, HZ can appear when a person is under a lot of stress. Here, the normal number of lymphocytes is opposed to a decrease in cellular-mediated immunity as a cause of the reactivation of shingles, an increase in psychological stress related to COVID-19 can explain its reactivated zoster. [8]. The median time to be diagnosed with COVID-19 and shingles was 5.5 days and acyclovir resolved the lesions after 10 days [2]. 

Anyone who has had a natural infection with wild-type varicella-zoster virus (VZV) or had varicella vaccination can develop herpes zoster. Children who get vaccinated have a lower risk of herpes zoster compared with children who were infected with wild varicella. The majority of people only have one episode in their lives, but repeated episodes are conceivable [9]. According to one study, herpes zoster is the underlying cause of 96 deaths each year (0.28 to 0.69 per 1 million population). Almost everyone who died was elderly or had a damaged or suppressed immune system. A live, attenuated vaccine that boosts the immunity to VZV and reduces the risk of HZ is now available and is recommended for adults older than 60 years of age. It has been known to reduce the incidence of HZ and PHN [2]. Even after so many recent advancements in the treatment modalities for PHN, it persists in many individuals influencing their daily activities and reducing their quality of life. Anticonvulsants, antidepressants, topical lidocaine, topical capsaicin, and options are the most widely used therapies for the treatment of PHN. One should be wary of the adverse effects of the drugs [10].

Herpes zoster has recently resurfaced, and the inactivated COVID-19 vaccine has been implicated, especially with CoronaVac which carries an inactivated portion of coronavirus. In addition, there was a 5-day latency period between the onset of herpes zoster and the administration of the inactivated COVID-19 vaccination [11]. Vaccines can also cause herpes zoster reactivation, as seen in patients who received inactivated hepatitis A, influenza, rabies, or Japanese encephalitis vaccines [12]. After yellow fever immunization, Bayas et al. reported a case of brachial plexus zoster [13]. Acute retinal necrosis is another consequence of varicella-zoster infection, according to Rothova et al [14]. Immune reconstitution inflammatory syndrome (IRIS), a paradoxical exacerbation of preexisting illness concealed by the host's renewed ability to mount an inflammatory response following the commencement of ART, may be analogous to vaccine-induced reactivation of HZ [15]. Vaccine trials do not include the person-time to estimate the incidence of rare events [16].

One of the most prevalent viral infections linked to facial palsy is HZ. In a comprehensive study of 1701 cases of Bell's palsy, 116 people (6.8%) were found to have HZ [17]. Furthermore, a portion of Bell's palsy patients have Ramsay Hunt syndrome (zoster sine herpete), which is diagnosed by a fourfold increase in VZV antibody or the detection of VZV DNA in auricular skin, blood mononuclear cells, middle ear fluid, or saliva [18].

## Conclusions

HZ is an important co-morbidity associated with COVID-19 which could severely affect the quality of life. It could also affect the treatment of COVID-19 since steroids that have established efficacy in COVID-19 cannot be used because of the risk of flaring up HZ. Due to the paucity of data regarding HZ in COVID-19 with no established guidelines to treat the condition it serves as an important part of literature and future research. Currently, analgesics and antivirals are the mainstays of treatment.
